# Regulation of Ack-Family Nonreceptor Tyrosine Kinases

**DOI:** 10.1155/2011/742372

**Published:** 2011-02-17

**Authors:** Victoria Prieto-Echagüe, W. Todd Miller

**Affiliations:** Department of Physiology and Biophysics, School of Medicine, Stony Brook University, Basic Science Tower T5, Nicolls Road, Stony Brook, NY 11794, USA

## Abstract

Ack family non-receptor tyrosine kinases are unique with regard to their domain composition and regulatory properties. Human Ack1 (activated Cdc42-associated kinase) is ubiquitously expressed and is activated by signals that include growth factors and integrin-mediated cell adhesion. Stimulation leads to Ack1 autophosphorylation and to phosphorylation of additional residues in the C-terminus. The N-terminal SAM domain is required for full activation. Ack1 exerts some of its effects via protein-protein interactions that are independent of its kinase activity. In the basal state, Ack1 activity is suppressed by an intramolecular interaction between the catalytic domain and the C-terminal region. Inappropriate Ack1 activation and signaling has been implicated in the development, progression, and metastasis of several forms of cancer. Thus, there is increasing interest in Ack1 as a drug target, and studies of the regulatory properties of the enzyme may reveal features that can be exploited in inhibitor design.

## 1. Introduction

 The mammalian nonreceptor tyrosine kinases (NRTKs) are divided into ten families: Src, Abl, Jak, Ack, Csk, Fak, Fes, Frk, Tec, and Syk [1]. In addition to their tyrosine kinase catalytic domains, they all contain noncatalytic domains that are important in enzyme regulation and substrate recognition [2]. Like all eukaryotic protein kinases, NRTK catalytic domains have an N-terminal lobe (N-lobe) that contacts ATP, and a larger C-terminal lobe (C-lobe). The activation state of the tyrosine kinases depends on the orientation of an alpha helix (*α*C) located in the N-lobe. In the active conformation, the *α*C helix projects inward toward the ATP-binding site [3]. The conformation of a flexible segment in the C-lobe (the activation loop) also has a key role in the regulation of the enzyme activity [4]. The regulatory importance of the phosphorylation of the activation loop varies in the different families of NRTKs. For instance, Src activation is strongly dependent on autophosphorylation [5], while Csk lacks the autophosphorylation site [6].

The contributions of the noncatalytic domains to enzyme regulation are well understood in the case of the Src-family of NRTKS. These enzymes have an N-terminal myristoylation site followed by an SH3 domain, an SH2 domain, and a kinase domain. Two tyrosine residues are important for their activity: Tyr 416 in the activation loop and Tyr 527 in the C-terminal tail. Structural studies showed the spatial arrangement of Src domains in the inactive state, in which the noncatalytic domains mediate intramolecular interactions that maintain the enzyme in a closed, inactive conformation. In this conformation, the SH3 domain is bound to a proline-rich helix located between the SH2 domain and the kinase domain, and the SH2 domain is bound to the phosphorylated Tyr 527 [7, 8]. Activating signals provided by protein-protein interactions that destabilize these intramolecular interactions may be of three types [2]: the binding of the SH3 domain to a proline-rich motif (SH3 binding domain) [9]; the dephosphorylation of the Tyr527 in the C-terminal tail [10]; the binding of the SH2 domain to other phospho-Tyr containing proteins [11]. These signals disrupt the intramolecular inhibition and activate the enzyme. In addition, the noncatalytic domains provide surfaces for different types of interactions that couple the activation of the catalytic domain to substrate recognition [2, 12, 13]. 

The mechanisms involved in enzymatic regulation vary among the different families of NRTKs. For example, Abl kinases have similar arrangements of domains as Src-family kinases, but the regulatory mechanisms are different; this has important implications for the design of pharmacological inhibitors [3]. In both Src- and Abl-family NRTKS, the SH3 domain interacts with a poly-proline helix in the linker region between the SH2 domain. In contrast to Src, the SH2 domain of Abl interacts in a phospho-Tyr independent manner with the C-terminal lobe of the kinase domain. An additional difference is that an N-terminal cap and a myristoyl group wrap around the base of the kinase domain of Abl and stabilize the inhibited structure [14]. Importantly, the conformation of the inhibited activation loop is unique to the c-Abl catalytic domain. The inhibitor Imatinib (Gleevec) has been particularly successful due to its ability to target this conformation [14].

As new structures of NRTKs are solved, new molecular mechanisms for enzyme regulation are revealed. Structural studies have illuminated the contributions of noncatalytic domains to the regulation of Syk- [3, 5], Csk- [6, 15–17], and Fes-family kinases [3]. The specific roles of the noncatalytic regions vary among these kinases. For instance, in Csk and Fes kinases, interactions between the SH2 and the catalytic domains stabilize the active conformation [3]. In general, the noncatalytic domains of all NRTKs are likely to participate in subcellular localization, autoregulation, and substrate targeting.

## 2. Ack Family Kinases

**Figure 1:**
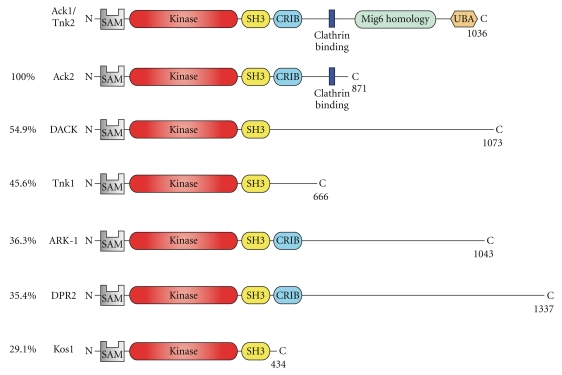
Ack family kinases. Domain arrangement of the members of the Ack family discussed in this paper. The different proteins are arranged in decreasing order of identity to the common core (Nt-Kinase-SH3).

The domain architecture of human Ack1 (also called Tnk2) is shown in Figure 1 (top). Ack1 is a 120 kDa protein with an N-terminal sterile alpha motif (SAM) domain [18], a kinase domain, an SH3 domain, and a Cdc42/Rac-interactive domain (CRIB) [19]. Several functionally relevant regions have been identified in the C-terminal region of Ack1: multiple proline-rich sequences, a clathrin-binding motif [20], an ubiquitin binding domain [21], and a region that shares a high homology with Mig6 [22]. The Ack family kinases have a unique domain arrangement. They are the only NRTKs with the SH3 domain located C-terminal to the kinase domain, and they are also the only tyrosine kinases with a CRIB domain. In view of its unique domain composition, the regulatory features of Ack1 are likely to differ from those observed in other NRTKs. 

Ack1 belongs to a family of NRTKs that includes human Tnk1, and homologous proteins in mouse, cow, *Drosophila melanogaster, *and *Caenorhabditis elegans * [23, 24]. Although all the members share the overall domain architecture shown in Figure 1 (i.e., kinase domain and SH3 domain), there are major differences in the C-terminal regions of the proteins. Furthermore, Tnk1, DACK (from *Drosophila*), and Kos1 (from mouse) lack CRIB domains [24].

Ack1, the first member of the Ack family, was cloned from a human hippocampal expression library by its ability to bind GTP-bound Cdc42 specifically and not Rac1 or RhoA [19]. The gene encoding Ack1 is located on chromosome 3q29 in humans, a region that is associated with recurrence of prostate cancer and is a predictor of metastatic relapse in breast cancer [25].

Tnk1 (thirty-eight-negative kinase 1) was originally cloned from hematopoietic stem/progenitor cells from umbilical cord blood in humans [26]. Tnk1 is a member of the Ack family with a predicted size of 72 kDa and the same domain architecture as Ack for the region common to both family members [26]. Kos1 is the murine homologue of Tnk1 [27]. In contrast with the other members of the Ack family (namely, Ack1/Tnk2, Ack2, and DPR2), but similarly to DACK, Tnk1, and Kos1 lack the CRIB domain [24, 27]. Tnk1, was found to be specifically expressed in umbilical blood and not other hematopoietic tissues and to interact with phospholipase C-*γ*1 through its proline-rich region [28]. It has been reported that during embryonic development, Tnk1 blocks NF-*κ*B activation and thereby promotes apoptosis mediated by the TNF*α* signaling pathway [29]. Kos1 is a 47 kDa protein that was cloned from differentiating murine embryonic stem cells [27]. The *Kos1* gene is located in chromosome 11 in mice and it is thought to be a murine splice variant of the human Tnk1 [27]. Tnk1/Kos1 have been reported to have proapoptotic or tumor-suppressor functions. Kos1 inhibits Ras activation and negatively regulates cell growth [27]. Tnk1/Kos1 knockout in mice results in an increased rate of tumor development [30].

Ack2 was first cloned from a bovine brain expression library. Ack2 has a predicted size of 83 kDa and contains the same basic domain structure as Ack1. Ack2 is thought to be an isoform of Ack1 that is generated by alternative splicing and is not encoded by a different gene [18]. The Ack2 C-terminal region contains a 15-residue insert and has 344 fewer amino acids than Ack1 [31]. In humans there is no evidence for the existence of an Ack2 homologue [18, 25]. However, a number of the experimental studies on Ack (activation pathways, biochemical properties, and biological roles) focused on Ack2, and they will be discussed throughout this paper. 

In *Drosophila*, there are two members of the Ack family: DACK and DPR2. The roles of DPR2 and DACK have not been dissected but they are thought to be different because DPR2 has a Cdc42-binding domain, while DACK lacks one. Cdc42 regulates the expression of DACK in *Drosophila* embryos [24]. The tyrosine kinase activity of DACK has been implicated downstream of Cdc42 in the pathway that leads to dorsal closure of the epidermis during embryogenesis [24]. In *C. elegans*, Ark-1 (A Ras-regulating Kinase 1), a cytoplasmic tyrosine kinase that is related to Ack1, acts as a negative regulator of EGFR signaling. In a yeast two-hybrid analysis, Ark-1 interacts with the Grb2 homologue, Sem5 [23].

The degree of conservation of the full-length sequences varies among the family members. The common core that is conserved in all of the Ack family members spans from the N-terminus to the SH3 domain. The amino acid identity of this core (compared with Ack1) ranges between 100% for Ack2 and 29% for Kos1 (Figure 1). The Ack1 kinase domain itself shares *≈* 40% amino acid identity with other nonreceptor and receptor tyrosine kinases. Phylogenetic analyses of human tyrosine kinase catalytic domains place Ack1 near epidermal growth factor receptor (EGFR) family kinases [32, 33]. As described below, Ack1 and EGFR may also share aspects of enzymatic regulation.

## 3. Activation Pathways

Expression of Ack1 has been demonstrated in several tissues in mammals, with the highest expression in spleen, thymus, and brain [18]. A number of stimuli, including EGF, PDGF [18], bradykinin [31], agonists of the M3 muscarinic receptor [34], and integrin-mediated cell adhesion [35, 36] promote phosphorylation and activation of Ack1.

Upon EGF stimulation, Ack1 phosphorylates and activates the guanine exchange factor Dbl. Ack1 interacts with the Rho-family GTPase Cdc42 and the adaptor protein Grb2 and these interactions are essential for this pathway [37]. The association of Ack1 with Grb2 has been shown in several studies through different approaches. *In vitro* studies showed that Ack1 associates with several SH3 domains, including the SH3 domain from Grb2 [38]. Ack1 association with multiple receptor tyrosine kinases such as Axl, LTK, and ALK is also dependent on Grb2 [39]. 

Ack is involved in integrin *β*1-mediated signaling pathways that modulate processes such as cell migration, cell adhesion, and cell spreading. Ack2 is present in a complex with integrin *β*1; it is activated by cell adhesion to a fibronectin-coated surface in a Cdc42-dependent manner and it activates the phosphorylation of Jnk [36]. In response to Cdc42 activation, Ack1 mediates the phosphorylation of p130Cas to promote cell migration [35, 40]. Ack1 is found in a complex with Cas, Crk, and Cdc42 in collagen-stimulated cells [35]. Cas appears to be an important adaptor protein downstream of Ack1 in these pathways. A signaling complex composed of Cdc42, Ack1, and Cas also participates in the regulation of melanoma cell spreading by melanoma chondroitin sulphate proteoglycan (MCSP), a proteoglycan that is expressed on the surface of melanoma cells [40]. The expression of Ack2 in HeLa cells increases cell migration in a mechanism that also involves p130Cas and CrkII [41]. Thus, one of the physiological functions of Ack1 appears to be related to the regulation of cell adhesion and cell migration. These studies also suggest that Ack1 activity is involved in the development of metastatic potential of transformed cells. 

Several studies have focused on the role of Ack1 in EGF receptor trafficking and dynamics. Ack1 is recruited to EGFR following EGF stimulation [21]. EGFR stability and recycling are regulated by interactions between Ack1 and multiple protein partners including ubiquitin [21], clathrin heavy chain [20, 42], and SH3PX1 [43, 44]. In HeLa cells, overexpression of Ack1 inhibits EGFR endocytosis and causes the accumulation of EGF in internal structures of endocytic origin, suggesting that Ack1 is involved in the intracellular sorting of activated EGFR [45]. The knock-down of Ack1 increases the rate of recycling ^125^I-EGF to the plasma membrane and reduces the degradation of ^125^I-EGF, suggesting that Ack1 promotes degradation of EGFR [45]. In Cos7 cells, overexpression of Ack1 promotes EGFR degradation by binding to the ubiquitinated EGF receptor [21]. Increased stability of Ack1 caused by a somatic mutation (S985N) in the ubiquitin binding domain (UBA) results in defective EGFR downregulation and sustained activation of downstream signaling [46]. 

In addition to the effect of Ack1 on receptor stability and dynamics, there is a reciprocal effect on Ack1 stability. After receptor stimulation by EGF, Ack1 is recruited to an EGFR complex [21, 47]. This complex promotes the activation of Ack1 [18] and EGFR degradation [21, 45, 46, 48] as well as Ack1 turnover [49, 50]. The EGF-induced degradation of Ack1 is signaled by ubiquitination [49, 50]. The details of this mechanism are still under study. In one report, the E3 Ubiquitin ligase Nedd4-2 was shown to ubiquitinate the UBA domain of Ack1 and induce its degradation by proteasomes [49]. In a second report, the E3 Ligase Nedd4-1 was demonstrated to ubiquitinate the Ack1 SAM domain, while the deletion of the UBA domain enhanced ubiquitination. In these experiments, the degradation of Ack1 was mediated by lysosomes rather than by proteasomes [50]. In both cases, the WW domains of the E3 ligases bound to a conserved PPXY motif in Ack1.

## 4. Biochemical and Regulatory Properties of Ack1

There is currently no crystal structure of full-length Ack1. The crystal structure of the isolated kinase domain of Ack1 has been solved in both phosphorylated and unphosphorylated forms. In these structures, the activation loop conformations are similar and do not occlude the substrate-binding site, suggesting that the phosphorylation of the activation loop may not play a dramatic stimulatory role [59]. The only other structural information available is for the isolated CRIB domain of Ack1 in a complex with Cdc42 [60]. In the absence of a structure of full-length Ack1, biochemical experiments have helped to reveal how each of the domains contributes to Ack1 function.

**Figure 2:**
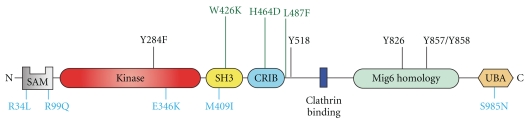
Residues important for Ack1 function. Phosphorylation sites are indicated in black. Tyr284 is the major site of autophosphorylation [38]. Tyr518, Tyr826, Tyr857, and Tyr858 were identified in proteomic studies [51–54]. Cancer-associated mutations are in blue [55]. Point mutations reported to affect Ack1 autophosphorylation are shown in green [18, 56, 57]. The numbering scheme used corresponds to the accession number Q07912.

A major autophosphorylation site has been mapped to the Y284 in the activation loop [38]. A construct containing residues 1- 476, corresponding to the N-terminus, the kinase domain, the SH3 domain, and the CRIB domain (Nt-Kinase-SH3-CRIB) is the longest construct that has been purified to homogeneity [38]. This construct is activated, although modestly (3-fold), by the phosphorylation of Y284. Proteomic approaches have identified other phosphorylation sites in Ack1. In BCR-ABL transformed cell lines, Y826, Y856, and Y857 of Ack1 are phosphorylated [51] (Figure 2). The phosphorylation of Y826 was also identified in a study of phosphorylated proteins downstream of insulin signaling [52]. Y857 of Ack1 was identified in a mass spectrometry study in which protein phosphorylation events downstream of the EGFR-family member HER2 were analyzed [53]. In addition, the phosphorylation of Y518 was identified in a study of global tyrosine phosphorylation patterns in cancer cells [54]. The biological significance of these phosphorylation sites remains to be determined. Interestingly, Y826, Y857, and Y858 are located in the Mig6-homology region (MHR), a portion of Ack1 that participates in inhibitory intramolecular interactions [58]. It is possible that the MHR of Ack1 is a substrate for oncogenic BCR-ABL, or for insulin and EGF receptors, resulting the release of autoinhibition. 

Relatively little information is available about the regulatory mechanisms of Ack1 and the roles that each domain plays in Ack1 regulation. The purified Nt-Kinase-SH3-CRIB polypeptide is active *in vitro* and can be used in enzymatic assays [38]. The peptide substrates that are the most efficiently phosphorylated by this construct are EAIYAAPFAKKKG (Abl consensus substrate) or KVIYDFIEKKKKG (peptide derived from WASP tyrosine phosphorylation site) [61]. In addition to this tyrosine kinase activity, Ack1 acts as a dual-specificity kinase towards WASP and the phosphorylation of a serine residue of WASP (Wiskott-Aldrich syndrome protein) increases the ability of WASP to polymerize actin [62]. Ligands for the SH3 domain and the CRIB domain bind to this construct, but fail to activate it, although addition of Cdc42 *in vivo* does increase Ack1 Y284 phosphorylation [38]. Residues important for the interaction between Ack1 and Cdc42 have been identified from the NMR structure of the complex formed by a peptide spanning the residues 548–489 of Ack1 (CRIB domain is 548–475) and Cdc42 [60]. 

One way of studying the role of each domain in Ack1 regulation is to assess the effects of point mutations on the enzyme's phosphorylation state (Figure 2). A mutation that occurs in ovarian endometrioid carcinoma (E346K) has been helpful to gain insight into the role of the Mig6 homology region (MHR). These studies have suggested that there is an autoinhibitory interaction between the C-lobe of Ack1 kinase domain and the MHR [58]. Mutations such as V365R (in the kinase domain) or F820A and E346K (in the MHR) destabilize this conformation and activate Ack1 [58]. Ack is activated by additional cancer-associated mutations located in the SAM domain (R34L and R99Q), and in the SH3 domain (M409I), but the mechanism is not clear. Several studies have produced a constitutively active Ack1 by the point mutation of L487F in the CRIB domain [56, 57]. The position of this mutation is thought to be analogous to L107F in Pak1, which is known to prevent an autoinhibitory interaction [63]. However, it is not known whether the CRIB domain of Ack1 participates in autoinhibition. A mutation designed to prevent the binding of the SH3 domain to its ligand (W426K) was reported to produce activation of Ack1 [18]. In contrast, a point mutation that disrupts Cdc42 binding (H464D) reduced Ack1 autophosphorylation [18]. 

**Figure 3:**
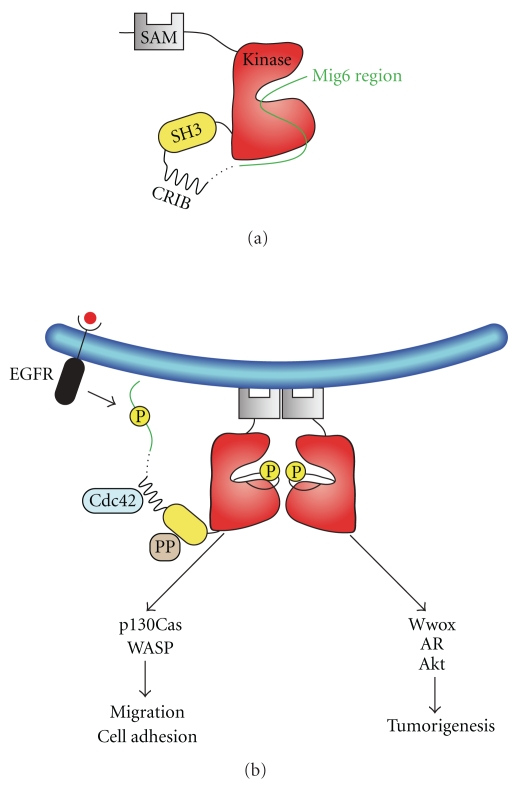
A model for Ack1 regulation. (a), the downregulated state of Ack1 is stabilized by an intramolecular interaction of the Mig6 homology region (MHR) with the kinase domain [58]. (b), the activated conformation is depicted when bound to the plasma membrane or an internal membrane. Stimulatory signals can be of three types: binding of GTP-bound Cdc42 to the CRIB domain, binding of poly-proline containing sequences (PP) to the SH3 domain, or phosphorylation of the MHR by activated receptors such as EGFR. The SAM domain is required for membrane localization and maximal activity. Upon activation, Ack1 phosphorylates downstream substrates such as p130Cas or WASP and modulates cell adhesion and cell migration. Ack1 promotes tumorigenesis by phosphorylating Wwox, Akt, and Androgen receptor. Yellow circles represent phosphorylation sites.

The deletion of the N-terminal SAM domain reduced Ack1 autophosphorylation and kinase activity [18, 64]. The SAM domain has the potential to drive Ack1 to the plasma membrane [18, 64] and to form dimers or multimers that increase the local concentration in order to stimulate Ack1 activation [64]. Interestingly, the deletion of the Ack1 SAM domain also prevented EGF-induced Ack1 ubiquitination, consistent with the requirement of the SAM domain for activation [64] and with the observation that Ack1 is ubiquitinated after activation [50]. These results suggest that the individual domains of Ack1 are involved in regulatory interactions that could be either inter- or intramolecular. The downregulated state of Ack1 involves the intramolecular interaction of the MHR with the kinase domain (Figure 3(a)). As a result of a stimulatory signal, provided by activated EGFR, one or more tyrosine residues located in the Ack1 MHR are phosphorylated (Figure 3(b)). These phosphorylation events may promote Ack1 activation by destabilizing the autoinhibited conformation. In addition, the SAM domain of Ack1 is required for full autophosphorylation and activation of Ack1. It is not clear whether the SAM domain participates in autoinhibition but as noted above, it is involved in subcellular targeting and required for maximal enzymatic activity.

The domain composition of Ack1 is complex and it is likely that in the future, additional interactions (both intra-and intermolecular) will be described that will explain in more detail the molecular mechanisms of Ack1 regulation and signaling.

## 5. The Role of Ack1 in Cancer

Ack1 has been implicated in several stages and several types of cancer; this topic has been reviewed recently in [65]. Early experiments demonstrated that a peptide derived from the Ack1 CRIB domain blocked the Ras-mediated transformation of NIH-3T3 cells [66]. Ack1 expression was later shown to be required for the survival of cells transformed by v-Ras [67]. 

In a study of ~180 advanced-stage cancers, 9% of the lung tumors and 14% of the ovary tumors had an amplification of the *Ack1* gene on chromosome 3. In addition, 42% of the aggressive lung tumors and 77% of metastatic hormone refractory prostate tumors showed overexpression of the Ack1 mRNA. Ack1 overexpression enhanced migration of human mammary epithelial cells (HMECs) and increased metastasis in the mouse mammary 4T1 breast cancer system [25]. Moreover, activation of Ack1 by overexpression of Ack1 produced several epithelial-to-mesenchymal transition (EMT) phenotypes in the human breast cancer cell line MDA-MB-231. In renal cancer cells, activation of Ack1 by a somatic point mutation also resulted in EMT phenotypes [46]. Thus, activation of Ack1 is associated with poor prognosis and metastatic phenotypes in human tumors [25]. In addition, the knockdown of Ack1 in breast cancer cells inhibits cell migration [47]. This suggests a role for Ack1 as a metastasis determinant, perhaps as a modifier of other pre-metastatic lesions.

The mechanistic basis of Ack1's involvement in prostate tumorigenesis has been explored in detail. In the cultured prostate cancer cell line LNCaP, the tumor suppressor Wwox is phosphorylated and marked for degradation by Ack1 [57]. The androgen receptor (AR) is a transcriptional activator that plays important roles in the development of advanced stage (androgen-independent) prostate cancer. Activated Ack1 has been shown to phosphorylate and activate AR function and to promote the progression of prostate cancer [68]. In addition, Ack1 Tyr284 phosphorylation was recently found to correlate with disease progression and proposed to be prognostic of progression of prostate cancer [69]. 

A large-scale study that screened cancer tissues for somatic mutations identified four missense mutations in Ack1 [55]. In lung adenocarcinoma and ovarian carcinoma, two mutations located in the N-terminus (R34L and R99Q, resp.) were identified; in ovarian endometrioid carcinoma, a mutation in the kinase catalytic domain (E346K) was identified; and in lung adenocarcinoma, a mutation in the SH3 domain (M409I) was found (Figure 2). The four cancer-associated mutations activate Ack1 and promote anchorage independent growth and cell migration [58]. An additional somatic mutation found in renal cancer, S985N, located in the UBA domain of Ack1, increases Ack1 stability and enhances oncogenic signaling through EGFR [46]. 

The functions of Ack1 in cancer cells appear to be of two types: kinase-independent and kinase-dependent. The effect of Ack1 on cell proliferation is independent of Ack1 activity [58]. In a phosphoproteomic study of the action of the antitumor drug dasatinib in lung cancer, Ack1 emerged as a signaling node that represents a possible target in cancer treatment. This study highlighted a possible scaffolding function of Ack1 in the context of control of cell growth [70]. In contrast, the activation state of Ack1 appears to be important in processes that involve enhanced cell motility, that are crucial in the metastatic stage of cancer progression and correlate with poor prognosis [25, 58, 67]. In breast cancer, Ack1 phosphorylates and promotes the activation of Akt, an important mediator of signaling pathways that lead to transformation [71].

## 6. Known Ligands and Substrates of Ack1

A number of Ack1 substrates have been identified in normal cells as well as cancer cells. Phosphorylation of WASP by Ack1 promotes its actin remodeling activity [62]. In addition, p130Cas is phosphorylated by Ack1, promoting cell spreading [40] and cell migration [35]. These results, together with the enhanced migration of cells overexpressing Ack1 [25] or expressing an activated form of Ack1 [58], suggest that Ack1 plays a role in the regulation of cell adhesion and migration. Ack1 also phosphorylates androgen receptor (AR), increasing the levels of phosphorylated AR in prostate cancer and promoting AR-mediated gene transcription [68, 69]. 

 A comprehensive list of known Ack1 substrates and interacting proteins was recently published [65], and it includes diverse molecules involved in signaling such as receptor tyrosine kinases (MERK, EGFR, PDGFR, AXL, ALK, and LTK), membrane proteins (integrin, MCSP), adaptor molecules (Grb2, HSH2), nucleotide exchange factors (Dbl), a transcription activator (AR), a tumor suppressor (Wwox), and a proto-oncogene (Akt/PKB). Other Ack1 interactors include proteins involved with vesicle dynamics (clathrin, SNX9) and cytoskeleton remodeling (WASP). A number of SH3 containing proteins, such as Hck [38], Grb2, and SNX9 [44, 72] have been identified as ligands for the proline rich region of Ack1. Recently, the E3 ubiquitin ligases Nedd4-1 and Nedd4-2 were shown to bind to a PPXY motif of Ack1 and to regulate its proteolytic degradation [49, 50].

## 7. Conclusions

Nonreceptor tyrosine kinases share several common features. The NRTK catalytic domains never occur in isolation; they are tethered to noncatalytic modular domains that play important roles in enzymatic function. The biochemical properties of the individual noncatalytic domains are similar to those found in other signaling proteins. However, the regulatory mechanisms are diverse and vary between the different families of NRTKs. To date, 15 out of the 32 NRTKs in the human genome have been shown to have oncogenic potential [1]. Consequently, NRTKs represent significant targets for the development of inhibitors. Activation of Ack1 (by overexpression or mutation) promotes migration and EMT and correlates with cancer progression. Thus, Ack1 has emerged as a candidate for anti cancer drug design; Ack1 inhibitors could potentially be used in combination therapies with inhibitors of other tyrosine kinases, such as EGFR. Knowledge about NRTK regulatory mechanisms can be exploited for the development of specific inhibitory drugs. For example, imatinib inhibits Abl (but not Src) because it binds to a specific conformation of Abl that Src is not capable of adopting [73]. Moreover, the inhibited conformation of Abl appears to be the result of the interplay of the noncatalytic domains [73]. Ack1 is regulated by a novel mechanism that shares basic principles with other NRTKs but also has unique features dictated by its unusual domain structure. Investigations into the regulatory mechanisms and conformational states of Ack1 will be an important prelude to the development of specific Ack1 inhibitors.
